# Genital Tract Infections in an Isolated Community: 100 Women of the Príncipe Island

**DOI:** 10.1155/2017/3058569

**Published:** 2017-11-13

**Authors:** Pedro Vieira-Baptista, Svitrigaile Grinceviciene, Gert Bellen, Carlos Sousa, Conceição Saldanha, Davy Vanden Broeck, John-Paul Bogers, Gilbert Donders

**Affiliations:** ^1^Department of Gynecology and Obstetrics, Centro Hospitalar de São João, Porto, Portugal; ^2^Ascendere Non-Governmental Organization, Lisbon, Portugal; ^3^Femicare, Clinical Research for Women, Tienen, Belgium; ^4^Vilnius University Institute of Biotechnology, Department of Biothermodynamics and Drug Design, Vilnius, Lithuania; ^5^Laboratório de Anatomia Patológica (LAP), Unilabs, Porto, Portugal; ^6^Algemeen Medisch Laboratorium, Antwerp, Belgium; ^7^Antwerp University Hospital, Antwerp, Belgium; ^8^Regional Hospital Heilig Hart Tienen, Tienen, Belgium

## Abstract

**Objective:**

To characterize the vaginal microbiome and the rate of sexually transmitted infections (STIs) in the women of Príncipe (São Tomé and Príncipe).

**Methods:**

Cross-sectional study of 100 consecutive women, invited for a free appointment and cervical cancer screening. A vaginal slide (wet mount microscopy) and a cervical sample (ThinPrep®) (Pap test, high risk* human papillomavirus* [HR-HPV],* N*.* gonorrhea* [NG],* T*.* vaginalis* [TV], and* C*.* trachomatis* [CT]) were obtained.

**Results:**

TV, NG, CT, and HIV were found in 8.0%, 2.0%, 3.0%, and 2.0%, respectively, and were more prevalent in younger women. HR-HPV was positive in 36.7%; 2 were positive for HPV18, but none for HPV16. Coinfection of HPV with other STIs was 8.3%. Prevalence of abnormal vaginal flora (AVF) was 82.5%, mostly bacterial vaginosis (BV) 54.6%, and moderate/severe aerobic vaginitis (msAV) 25.8%. HR-HPV was not related to BV (*p* = 0.67). The association of abnormal Pap test with msAV was not significant (*p* = 0.08).

**Conclusion:**

The prevalence of NG, CT, TV, and HR-HPV was according to expected, while that of HR-AVF was higher. The surprisingly low prevalence of HPV16 and HPV18 must be considered in the design of programs for prevention and vaccination; this setting can be useful as a model for postvaccination scenarios.

## 1. Introduction

Sexually transmitted infections (STIs) constitute a worldwide, major public health problem [1]. Detailed information about the prevalence of STIs is especially important to enable better development of primary and secondary prevention models. This is even more important in low-resource countries, where limited access to medical care promotes a rapid and widespread of infections and, as a consequence, more complications and unfavorable pregnancy outcomes. With increasing prevalence, the likelihood of coinfection increases, which hence is an indicator of behavioral habits [2].

The prevalence of STIs differs between regions.* Trichomonas vaginalis* (TV) prevalence is highest in African and American regions, and the lowest rates are reported in the Western Pacific region [3].* Chlamydia trachomatis* (CT), on the other hand, has its highest prevalence in North and South America, while in Africa it is only the fourth most frequent STI [3].* Neisseria gonorrhea* (NG) prevalence is high in Africa and in the Western Pacific region, but low in the American continent [3]. Prevalence of human immunodeficiency virus (HIV) is 4.7% in Africa but differs strongly from country to country, mounting up to 24% among women in some regions [4]. Several other conditions also play a role: in some African countries (South Africa and Zimbabwe), for instance, pregnant women were at increased risk of acquiring an STI due to decreased condom use [5].

Not much is known about sexual habits, STIs, and risk factors of Príncipe Island's community or how it compares with the rest of the sub-Saharan regions. The aim of our research is to investigate the presence of STIs and the risk factors among a limited sample of women of the isolated community of Príncipe Island, São Tomé and Príncipe, in order to identify the most urgent needs and plan subsequent actions accordingly.

## 2. Materials and Methods

A cross-sectional observational study was conducted at Príncipe Island, São Tomé and Príncipe, in 2015. To get an idea of the prevalence of abnormal vaginal flora (AVF) and sexually transmitted infections (STIs), 100 women were invited (radio, religious services, word of mouth) to attend a free appointment, including a Pap test. The present study was approved and authorized by a local ethical committee. The samples were taken at the local hospital, in which only very basic needs are provided. All women consented in participating in the screening project before sample collection. A questionnaire was filled, and samples were collected for cytology, vaginal wet mount examination, and nucleic acid amplification test (NAAT) testing for* NG*,* CT*,* TV*, and HPV testing. Patients were given advice and received treatment for any STIs diagnosed. In case of abnormal Pap smear findings, colposcopy and cervical biopsies were performed during a following visit. Women's HIV status is known by population, as testing is routinely and frequently performed.

Vaginal smears were taken through a speculum from the upper vaginal wall and spread evenly on glass slides, air dried, and transported for later reading examination [6]. Magnification of 400 times and phase contrast microscope were used for readings. According to Femicare's systematic reading method, the following types of lactobacilli grade (LGB) were identified: (I) numerous pleiomorphic lactobacilli, no other bacteria; (IIa) mixed flora, but predominantly lactobacilli; (IIb) mixed flora, but proportion of lactobacilli severely decreased due to increased number of other bacteria; (III) lactobacilli severely depressed or absent because of overgrowth of other bacteria [6]. Slides were also classified as* Candida* colonization (observing hyphae, pseudohyphae, or blastospores); partial BV (mixed lactobacilli, grade (IIa) or (IIb) with areas of other anaerobic flora; sporadic clue cells, less than 20%) and full BV (if granular flora is covering all areas of the slide and >20% clue cells); aerobic vaginitis (AV) (diagnosed according the composite score [7] of 5 variables: lactobacillary grade, proportional number of leucocytes, proportion of toxic leucocytes, proportion of parabasal cells, and presence of aerobic flora; the scoring system of each findings was published elsewhere [7]). If the attained AV score was equal or superior to 5, moderate or severe AV (msAV) was diagnosed. The microscopy findings were identified according to standardized protocol [8] and confirmed in a second reading by two microscopists (GB, SG). Both microscopists were blinded to all other patient data.

A liquid-based Pap smear sample was obtained from all women using a cervical brush to perform cytology and polymerase chain reaction (PCR) to detect sexual transmitted infections. HPV genotyping was performed using the Abbott Real-Time High Risk HPV assay for 16 and 18 and for the other 12 high risk types (31, 33, 35, 39, 45, 51, 52, 56, 58, 59, 66, 68). Viral DNA was extracted, amplified by qPCR, and partially genotyped according to the manufacturer's instructions; negative and positive control samples were included in each analysis.

Statistical analysis was performed using SPSS version 23 (IBM, Armonk, NY, USA). Patients were divided into two age groups, till 29 years old and 29 years and older. Values were expressed as numbers and percentages and the Fisher exact test was used to compare results. Mann-Whitney *U* test was used for two independent continuous variables comparison. A *p* value < 0.05 was considered statistically significant.

## 3. Results

Main characteristics of the 100 participating women are presented in Table 1. One-fifth of the women (23.0%) were pregnant and 9% were menopausal. One-third did not use any contraception, 2 had tubal ligation, 15 used injectable medroxyprogesterone acetate, 10 were on oral contraception, 3 had a subdermal implant, and 1 had an intrauterine device. Only one woman used condoms, and 2 failed to report on contraception choice.

The age range was 21–60 years (mean 34.8 ± 0.71). The reported mean age at coitarche was 17.5 ± 2.8 years and the number of sexual partners ranged between 1 and 8 (mean 2.5 ± 2.8). Mean number of abortions was 0.6 ± 0.9. Age at first delivery ranged between 14 and 32 years with a mean of 19.5 ± 2.9 years. Women delivered up to 11 children, with a mean of 3.5 ± 2.3. Only four women were nulliparous.

Prevalence of TV was 8.0%, NG 2.0%, and CT 3.0%. One woman had both NG and TV and one NG and CT. Two women were HIV positive. One-third of the women (*n* = 31) were <29 years old. There was a clear trend towards more STIs (CT, NG and TV) in younger women (*p* = 0.03), but numbers were too low to show significant differences individually (Table 2).

An error occurred in the HPV testing in 2 women. Overall prevalence of HPV positivity was 36.7% (36/98). Only 2 women had HPV18 and none had HPV16. None of patients with HPV was coinfected with CT, one was with NG and two with TV. So, coinfection of HPV with other STI was present in 3/36 (8.3%) HPV positive women. Eight women with HR-HPV infection (22.2%) had abnormal cervical cytology, but none of them was infected with CT, NG, or TV. There were no differences in STI rates between pregnant and nonpregnant women (Table 3).

Wet mount microscopy could not be performed in 3 women due to bad quality of the smear. 17.5% of women had LGB (I), 10.3% LBG (IIa), 21% LBG (IIb), and half (49.5%) LGB (III) (no lactobacilli). Prevalence of abnormal vaginal flora (LGB (IIb) and LGB (III)) was 71.0% (69/97), of full blown BV 34.0% (33/97) and of partial BV 20.6% (20/97). Severe AV was found in 9 (9.3%), moderate AV in 16 (16.5%), and light AV in 20/97 (20.6%) women.* Candida* was found in 4.1% (4/97) of women. Normal flora (absence of BV, AV, or* Candida*) was encountered in only 14.4% (14/97) of women. There was a trend towards a more frequent finding of msAV among HR-HPV positive women (*p* = 0.085), but no differences were found for BV (*p* = 0.7).

Seven of 8 women with abnormal cytology (LSIL [*n* = 5], HSIL [*n* = 2], ASC-US [*n* = 1]) were HR-HPV positive. In 1 woman with HSIL we failed to get HR-HPV results due to technical difficulties. One of the HIV positive women had HR-HPV, but both had normal cytology.

There was no difference between STI rates according to age, menopause status, alcohol consumption, or parity, but women with STIs had a higher number of sexual partners (Table 3).

## 4. Discussion

In the present paper we report on the unique community of Príncipe Island, São Tomé and Príncipe, situated in the Gulf of Guinea To our knowledge, no STI prevalence data has been published so far. It is a community of about 7.000 inhabitants with limited access to the main island, mainland, or other areas of the world, as air travel is not affordable for the majority of the population and boat trips are time consuming. More than half of its population, in 2015, lived below national poverty level and the human development index in 2014 was only 0.555 [9], ranking it 166th out of 189 countries in terms of economy [10]. Due to this isolation, this island offers unique opportunities to study the presence of STIs, before globalization and changes in migration patterns may induce a shift towards more global trends of the presence of different STIs. In a similar setting, the most prevalent HPV serotypes were types 31, 33, 56, and 55 in an isolated population of American Indians [11, 12].

The prevalence of cervical HR-HPV infection in the first 100 evaluated women of this island was 33.7%, which is higher than what is common in most places, but similar to other Eastern sub-Saharan African countries (33.6%, CI 30.3–37.1) [13]. In the African continent, the most prevalent HPV genotypes have been invariably 16 and 18 [13], at least before the vaccination era. Similarly, HPV16 is the most prevalent serotype in other parts of the world, such as Germany [11], USA [14], Brazil [15], or Tunisia [16]. However, in our sample these types were not at all (HPV16) or very rarely (HPV18) detected. Fischer et al. (2016) found HPV16, HPV18, HPV31, and HPV42 to be suppressed in a vaccinated society [11], but as vaccinations have never been introduced in Príncipe, this cannot have had any role in our findings.

Prevalence of TV in Príncipe Island is lower than in mainland Southern Africa, where up to 25% (95% CI, 17.9–31.4) of women seem to be infected [17]. However, that TV prevalence is still higher than in Europe, where it ranges from 1.3% (Italy) [18] to 0.4% (Belgium) [19], and Latin America (3.6%, 95% CI: 2.2–5.6). In Mexico, the prevalence of TV was related to economical fluctuation: its frequency was found to be countercyclic and the authors related the finding to accessibility to healthcare service [20]. Our findings did not support this hypothesis, because São Tomé and Príncipe is one of the poorest countries in the world, and medical care is scarce. Still, the prevalence of TV is lower than in the African continent, while the prevalence of NG (2%) and CT (3%), in our sample, resembles those in neighboring African regions. CT prevalence is around 4% in most African and European regions, around 5.5% in the Americas, and lowest in Southeast Asia (1%), while NG prevalence, being typically dependent on the economic burden [20], is higher in Africa (2%) than in Europe (0.4%) [3]. A larger sample and longitudinal observations would be advantageous for a more accurate conclusion. In Brazil, Wohlmeister et al. found, in 2016, a lower prevalence of NG (1.2%), CT (9.5%), and TV (4.7%) than we did [21]. Nevertheless, in the same country, but 4 years before, de Abreu et al. (2016) found higher rates (NG 4.7%, CT 10.3%, and TV 11.6%) [22]. However, NG was also found to have cyclic relation to economic fluctuation in Mexico and became lower when economics went down [20]. For better comparison of the prevalence, economic factors should therefore be taken into account [20].

Despite the small size of the sample, we performed an analysis of the risk factors for acquiring STIs, such as condom use, pregnancy, and alcohol consumption. Only one woman used condoms, explaining a similar prevalence of STIs in pregnant and nonpregnant women. In Zimbabwe, lower use of condoms was associated with increased sexual risk behavior and higher pregnancy rates [5]. Sex without condom in the past three months was practiced by two-thirds of women in Zimbabwe, indicating condom use is not very popular [5]. Another factor which is related to a higher incidence of STIs is alcohol consumption [23], but we failed to confirm a relation between drinking habits and STIs. As smoking habits were not reported, we could not analyze its relation to STIs. Young age and high number of sex partners are clear risk factors for acquiring STI's, but not HR-HPV, in this population.

Unlike other researchers, we did not find an association between the presence of NG, CT, or TV and HPV or abnormal Pap smear findings [21, 22]. All tested women in Belgium with TV and HSIL also had HR-HPV, as well as more than half (59%) of women with TV and ASC-US [19]. Among HR-HPV negative women, TV was found in 1.3% of women with ASC-US, but only in 0.03% of women with normal cytology (OR 4.2, CL 95% 2.1–8.6) [19]. Donders et al. hence concluded that the copresence of TV and HR-HPV increased the likelihood of cytological abnormalities (*p* < 0.05), mainly due to an increase in ASC-US and LSIL, but not HSIL [19]. The size of the study of Príncipe Island did not allow conclusions of the relation between HPV or abnormal cytology and STIs and should be reassessed in larger samples.

Besides the high prevalence of HR-HPV other than HPV16 or HPV18, another major finding of this study was the staggering prevalence of AVF and vaginal infections. Apart from BV, which is found to be highly prevalent in most sub-Saharan African countries [24], the prevalence of AVF and AV has not been intensively studied in Africa yet. In South Africa and Uganda, however, it was clearly shown that AVF and AV are more frequent than in other parts of the world [25, 26] and presumably account for many of the potential side effects and STI transmission risk factors allegedly accounted to BV in several former studies [24, 27]. We currently did not find a clear association between cytology/positive HR-HPV and disrupted flora types such as AV or BV as Jahic, Vieira-Baptista, and Donders found [28, 29], but this can most likely be accounted to small numbers and pleas for more extensive research.

The fact that free cervical cancer screening was being offered might have been a bias. However, the main limitation of this study is its small sample size, precluding sophisticated (multivariate) analysis to be performed, but given the difficulty to gather the samples, and the naïve status of this remote, isolated area, we decided to start off with an in depth analysis of a small group of women first, to get an idea of the prevalence of the most common STIs in general. Further study of a larger contingent of this population to confirm specific trends will be needed to direct further actions and measures. Hence, we aim to subtype the different HPV subgenera in this population to obtain a clearer picture of the precise HPV subtype prevalence in this low HPV16/HPV18 prevalent area and decide whether and if vaccination with a broad spectrum HPV vaccine would be achievable and wise. Further research is also necessary, in order to improve the vaginal microflora, which is one of the riskiest influencing factors for acquiring HIV and other STIs, as well as for negative obstetrical outcomes worldwide. Certainly, it would have a tremendous impact in an isolated community which will, inevitably, one day open up for influxes from the rest of the world. In Uganda we recently achieved a significant improvement of the vaginal microflora by self-screening and treatment with locally applied intravaginal antiseptics or antibiotics [30].

## 5. Conclusion

Women from Príncipe Island have a very high prevalence of high risk AVF and HR-HPV, but not HPV16/HPV18. Despite that, the prevalence of classical sexually transmitted infections, such as NG, CT, and TV was similar to that in continental African regions. Prevalence data from isolated communities can be especially interesting for clinicians and policy makers, to tailor prevention and vaccination programs. Furthermore, the specific HPV prevalence characteristics in this population provide a platform for research to resolve scenarios that will ensue in the post HPV-vaccination with less or no HPV16 and HPV18 and potentially more other HR-HPV types.

## Figures and Tables

**Table 1:** Epidemiology of 100 subjects undergoing first Pap smear in Príncipe Island.

Characteristics	*N*	Mean ± SD	Median	Minimum	Maximum
Age	10	34.79 ± 9.71	32.0	21	60
Coitarche	91	17.53 ± 2.79	17.0	13	30
Age of first delivery	82	19.52 ± 2.88	19.0	14	32
Number of sexual partners	89	2.52 ± 1.40	2.0	1	8
Number of pregnancies	93	4.35 ± 2.36	4.0	0	11
Number of deliveries	93	3.49 ± 2.17	3.0	0	11
Number of abortions	93	0.61 ± 0.94	0.0	0	5

**Table 2:** STIs and microbiome changes in population in 100 unselected women of Príncipe Island. For BV, Candida and AV data were only present for 97 women.

Condition/age	<29 years (*n*, %)	≥29 years (*n*, %)	*p* value
	Total = 31	Total = 69	
*C. trachomatis* infection	2 (6.4%)	1 (1.5%)	0.23
*N. gonorrhea* infection	2 (6.4%)	0	0.09
*T. vaginalis* infection	5 (16.1%)	3 (4.3%)	0.10
*Any of above infections*	7	4	0.03
HR-HPV infection	10 (32.2%)	26 (37.7%)	0.82
HIV infection	0	2 (2.9%)	1.00
Bacterial vaginosis	14 (45.2%)	39 (56.5%)	0.39
*Candida*	2 (6.4%)	2 (2.9%)	0.59
Aerobic vaginitis	7 (22.6%)	18 (26.1%)	0.80

**Table 3:** Epidemiology and risk factors for STIs (HPV, HIV, CT, TV, or NG positive) of 100 unselected women presenting for Pap smear in the remote isolated population of Príncipe Island. Pearson *χ*^2^ Fisher exact test and Mann-Whitney *U* test were used.

Characteristic	No STIs	STIs present	*p* value
	*N* = 53	*N* = 47	

Menopause (%)	4 (7.5%)	5 (11.4%)	0.90
Alcohol use (%)	36 (67.9%)	26 (59.1%)	0.09
Pregnant (%)	14 (26.4%)	9 (19.1%)	0.50
Age (mean ± SD)	35.1 ± 9.3	34.7 ± 10.3	0.70
Coitarche (mean ± SD)	18.0 ± 3.1	17.1 ± 2.3	0.10
Age of first delivery (mean ± SD)	20.1 ± 3.1	18.9 ± 2.4	0.06
Number of sexual partners (mean ± SD)	2.3 ± 1.4	2.8 ± 1.4	0.03
Number of deliveries (mean ± SD)	3.4 ± 1.9	3.6 ± 2.5	0.90
Number of abortions (mean ± SD)	0.51 ± 1.0	0.74 ± 0.9	0.40

## Abbreviations and Acronyms

AML: Algemeen Medisch Laboratorium
ASC-US: Atypical cells of undetermined significance
AV: Aerobic vaginitis
AVF: High risk abnormal vaginal flora
BV: Bacterial vaginosis
CT: * C. trachomatis*
CV: * Candida* vaginitis
DNA: Deoxyribonucleic acid
HIV: Human immunodeficiency virus
HR-HPV: High risk* human papillomavirus*
HSIL: High-grade squamous intraepithelial lesion
IBM: International Business Machines Corporation
LGB: Lactobacilli grade
LSIL: Low-grade squamous intraepithelial lesion
msAV: Moderate/severe aerobic vaginitis
NAAT: Nucleic acid amplification testing
NG: * N. gonorrhea*
NY: New York
OR: Odds ratio
Pap smear: Papanicolaou smear
PCR: Polymerase chain reaction
qPCR: Quantitative polymerase chain reaction
SPSS: Statistical package for the social sciences
STIs: Sexually transmitted infections
TV: * T. vaginalis*
USA: The United States of America
*χ*^2^: Chi square test.
